# Serum Total SOD Activity and SOD1/2 Concentrations in Predicting All-Cause Mortality in Lung Cancer Patients

**DOI:** 10.3390/ph14111067

**Published:** 2021-10-21

**Authors:** Katarzyna Beata Skórska, Sylwia Płaczkowska, Anna Prescha, Irena Porębska, Monika Kosacka, Konrad Pawełczyk, Katarzyna Zabłocka-Słowińska

**Affiliations:** 1 Department of Food Science and Dietetics, Wroclaw Medical University, ul. Borowska 211, 50-556 Wroclaw, Poland; anna.prescha@umed.wroc.pl (A.P.); katarzyna.zablocka-slowinska@umed.wroc.pl (K.Z.-S.); 2 Diagnostics Laboratory for Teaching and Research, Department of Laboratory Diagnostics Wroclaw Medical University, ul. Borowska 211a, 50-556 Wroclaw, Poland; sylwia.placzkowska@umed.wroc.pl; 3 Department and Clinic of Pulmonology and Lung Cancers, Wroclaw Medical University, ul. Grabiszynska 105, 53-439 Wroclaw, Poland; irena.porebska@umed.wroc.pl (I.P.); monika.kosacka@umed.wroc.pl (M.K.); 4 Lower Silesian Centre of Lung Diseases, ul. Grabiszynska 105, 53-439 Wrocław, Poland; konrad.pawelczyk@umed.wroc.pl

**Keywords:** lung cancer, SOD1, SOD2, serum, total SOD activity, Glasgow prognostic score, albumin, C-reactive protein

## Abstract

Redox status disturbances are known during carcinogenesis and may have influence on patients’ survival. However, the prediction of mortality in lung cancer patients based on serum total SOD activity, and concentrations of its isoforms, has not been studied to date. This prospective cohort study has following aims: (1) to evaluate the disturbances in serum SOD activity and SOD1/2 concentrations; (2) to assess the implications of these alterations with regard to biochemical variables and clinical data, and (3) to investigate the association between serum SOD activity, SOD1/2 concentrations, and all-cause mortality in lung cancer patients. Serum total SOD activity and SOD1, SOD2, albumin, CRP, and ceruloplasmin concentrations were determined in lung cancer patients (n = 190) and control subjects (n = 52). Additionally, patients were characterized in terms of biochemical, clinical, and sociodemographic data. Multiple Cox regression models were used to estimate the association between all-cause death and SOD-related parameters. All-cause mortality in lung cancer was positively associated with serum SOD1 and SOD2 concentrations. Clinical stage III and IV disease was the strongest predictor. The utility of the evaluated parameters in predicting overall survival was demonstrated only for SOD1. Serum SOD1 and SOD2 concentrations were shown to positively affect all-cause mortality in lung cancer patients, but SOD1 seems to be a better predictor than SOD2.

## 1. Introduction

Recent global cancer statistics indicate that lung cancer is still the most commonly diagnosed cancer among men in countries of low socioeconomic status. Additionally, it remains the most common cause of oncological deaths, regardless of sex and economic status [1,2]. Currently, the average five-year survival rate for lung cancer patients in the United States is reported to be approximately 17% [3], and that in Europe is approximately 15% [2]. As lung cancer remains the leading cause of cancer-related death worldwide [2], the identification and availability of prognostic markers is important in the management of patients in order to increase survival outcomes.

The low survival rate of lung cancer is associated with its long asymptomatic course, which allows it to remain undetected until it is at an advanced stage [3]. The utilization of early prognostic biomarkers can markedly increase the efficiency of therapy, detection of recurrence, and enrollment of patients into different treatment regimens [4,5]. However, the presently employed methods for the diagnosis of lung cancer are not sufficiently effective [3,6]. 

Nearby 90% of lung cancer deaths are closely associated with smoking, with non-smokers accounting for only ~10–15% of all lung cancer patients [3]. The link between cigarette smoking and redox imbalance in lung cancer has been extensively studied, and is widely known [6,7,8]. Additionally, the lungs are vulnerable to increased endogenous and exogenous oxidative insults [9]. According to several studies, lung carcinogenesis is directly linked with redox imbalance—both locally and systemically expressed [7,10,11,12]. Oxidative stress is a cellular phenomenon or condition that occurs as a result of a physiological imbalance between the levels of antioxidants and oxidants (free radicals or reactive species), in favor of oxidants [13]. It can be also concluded that factors other than tobacco smoke may contribute to oxidative stress in lung cancer patients, since the prevalence of the disease is also systematically increasing among non-smokers [14]. Additionally, not all, but only a small percentage of individuals exposed to tobacco smoke develop lung cancer [15]. This may be partly because the body possesses a complex endogenous enzymatic and non-enzymatic antioxidant defense grid. The detoxification enzyme that acts as a component of the first-line defense system against reactive oxygen species (ROS) is superoxide dismutase (SOD) [9,16]. SOD catalyzes the dismutation of two molecules of the superoxide anion (*O_2_) to molecular oxygen (O_2_) and hydrogen peroxide (H_2_O_2_) which, in turn, are detoxified by catalase and glutathione peroxidase. Consequently, the potentially harmful superoxide anion becomes less hazardous [16].

SOD is a metalloenzyme, and hence requires a metal cofactor for its activity. In humans, there are three forms of SOD: superoxide dismutase 1 (SOD1, Cu/Zn-SOD), present in the cytoplasm; superoxide dismutase 2 (SOD2, Mn-SOD), located in the mitochondria, and extracellular superoxide dismutase 3 (SOD3, Cu/Zn-SOD) [17].

The data concerning the expression and activity of SOD, SOD1, and SOD2 in blood and tumor cells are ambiguous. Total SOD activity has been shown to be increased in lung carcinoma cells compared with tumor-free lung tissue [9]. Similarly, in the study of Kaynar et al. [18], erythrocyte SOD1 activity was significantly higher in patients with lung cancer than in the control group. Conversely, in another study, the SOD2 activity determined in the leukocyte cells of lung cancer patients was lower than that of the control group [19]. Additionally, one meta-analysis strongly suggests that changes in SOD2 activity associated with C47T polymorphism have an impact on the risk of lung cancer development [20]. Other investigations have shown that increased cell SOD2 activity facilitates cell migration and invasiveness of tumors [21,22]. However, no research on disturbances in serum SOD1 and SOD2 concentrations has been performed in lung cancer patients.

Considering other types of cancer, the SOD2 protein concentration and gene expression in esophageal cancer tissue were found to be decreased compared to in normal tissue [23], and pancreatic cancer cell lines had lower levels of SOD2 than normal human pancreas cells [24]. Follow-up and observational studies have suggested that SOD may protect against oxidative damage and extend lifespan in cancer patients [9,25], but data on this important subject are scarce. Therefore, the role of SOD in predicting mortality in cancer patients—especially lung cancer patients—requires further investigation.

The aims of this prospective cohort study were as follows: (1) to evaluate the changes in serum SOD activity, as well as SOD1, and SOD2 concentrations; (2) to assess the implications of these alterations with regard to biochemical variables and clinical data, and (3) to investigate the association between serum SOD activity, SOD1/2 concentrations, and all-cause mortality in patients with lung cancer.

## 2. Results

### 2.1. Baseline Characteristic of the Study Group

The baseline characteristics of the lung cancer patients and the control group are presented in Table 1. The comparison of the groups’ smoking habits showed that a significantly lower percentage of lung cancer patients had never smoked (23.2 vs. 53.8%), while a significantly higher number were former smokers (48.6 vs. 19.2%). A higher percentage of the control group consumed alcohol regularly (76.9 vs. 50.3%). The two groups did not differ in terms of sex distribution, level of education, passive smoking status, number of cigarettes smoked daily, or number of units of alcohol consumed weekly.

Differences in the values of biochemical parameters were also noted. The median serum SOD1 and SOD2 concentrations were significantly higher in lung cancer patients compared to control subjects (218.9 vs. 141.0 pg/mL, and 1.30 vs. 0.78 ng/mL, respectively). However, no significant differences were observed in serum total SOD activity between lung cancer patients and control subjects. Furthermore, serum albumin concentration was significantly lower in lung cancer patients than in control subjects (3.86 vs. 4.27 g/dL), and significantly more lung cancer patients than control subjects had albumin concentrations <3.5 g/dL (18.2 vs. 4.0%) indicating malnutrition. The reverse was observed in serum C-reactive protein (CRP) (11.24 vs. 2.26 mg/L) and ceruloplasmin (0.26 vs. 0.21 g/L) concentrations, and a greater percentage of lung cancer patients had CRP concentrations >10 mg/L compared to the control group (53.4 vs. 6.0%), indicating a higher prevalence of systemic inflammation. As a result of higher CRP concentrations and hypoalbuminemia, significantly more patients with lung cancer than subjects from the control group had a high Glasgow prognostic score (GPS).

Lung cancer patients were diagnosed and enrolled in this study at different clinical stages of the disease, and the majority of them were at clinical stage I (41.4%) and suffered from non-small-cell lung cancer (NSCLC) (94%). Post-study oncological treatment was as follows: about 70% of patients underwent lung cancer resection, 32.3% of subjects received chemotherapy, and 4.3% received radiotherapy. Among all of the patients, ~45% had been diagnosed with cardiovascular disease (CVD), 14% with chronic obstructive pulmonary disease (COPD), and 18% with diabetes mellitus (DM) type 1 or 2. Additionally, ~40% of lung cancer patients suffered from iron-deficiency anemia, 11% presented thrombocytopenia (<150 × 10^3^ cells/µL), and 7% had reactive thrombocytosis (>400 × 10^3^ cells/µL). Median phosphatase alkaline activity was ~80 U/L among lung cancer patients. More than one-third of patients had a decreased estimated glomerular filtration rate (eGFR), and ~7% of patients had serum creatinine levels >1.2 mg/dL, possibly indicating a failure of renal function. Moreover, elevated serum glucose concentrations (≥100 mg/dL) were found in more than half of patients.

To evaluate the implications of changes in parameters related to SOD with regard to the biochemical and clinical data of lung cancer patients, we compared the following parameters: albumin, CRP, ceruloplasmin, hemoglobin (Hgb), platelets, creatinine, glucose concentrations, neutrophil:lymphocyte ratio (NLR), eGFR, and phosphatase alkaline activity—as biochemical parameters—and the clinical stage of disease, presence of CVD, COPD, DM, anemia, reactive thrombocytosis, systemic inflammatory state, and elevated GPS—as clinical parameters—between subgroups of patients below and above the medians of certain parameters of SOD. Neither biochemical parameters nor clinical data differed between subgroups of lung cancer patients, except for lower concentrations of ceruloplasmin being found in lung cancer patients with SOD1 concentrations below the median (data are shown in Supplementary Table S1).

### 2.2. Cutoff Points of Serum Total SOD Activity and SOD1/2 Concentrations for the Diagnosis of Lung Cancer, Identification of Clinical Stage IV, and Prediction of Mortality in Lung Cancer Patients Using ROC Curves

Table 2 shows specific cutoff points, areas under the ROC curve (AUCs), and Youden’s indices for serum total SOD activity as well as SOD1 and SOD2 concentrations in the diagnosis of lung cancer, identification of clinical stage IV of the disease, and the prediction of mortality in lung cancer patients. 

Only serum SOD1 concentration significantly differentiated lung cancer patients from control subjects, with a cutoff value of 175.03 pg/ml, an AUC of 0.684 (0.608–0.759), and a Youden’s index value of 31%. Serum total SOD activity and SOD2 concentration did not significantly distinguish lung cancer patients from control subjects. Additionally, neither serum total SOD activity nor serum SOD1 and SOD2 concentrations were found to be useful indicators in determining clinical stage IV lung cancer. However, serum SOD1 and SOD2 concentrations significantly predicted mortality in lung cancer patients. The cutoff values of serum SOD1 and SOD2 concentrations that best differentiated survival and non-survival patients were 187.59 pg/Ml and 2.30 ng/mL, respectively, with AUCs (95% CI) of 0.654 (0.576–0.733) and 0.618 (0.530–0.705), (both *p* < 0.05) and Youden’s index values of 24 and 19%, respectively.

### 2.3. Associations between Serum Total SOD Activity, SOD1 and SOD2 Activity, and All-Cause Mortality in Patients with Lung Cancer

Table 3 presents significant factors predicting all-cause mortality in lung cancer patients. In the univariate Cox analysis, increased serum SOD1 concentration per 1 pg/mL, increased serum SOD2 concentration per 1 ng/mL, increased albumin concentration per 1 g/dL, CRP > 10 mg/dL, GPS > zero, serum ceruloplasmin concentration above the median value, clinical stages III and IV, presence of DM, platelets >400 × 10^3^ cells/µL, NLR above the median value, increased alkaline phosphatase activity per 1 U/L, and increased glucose per 1 mg/dL were all significant predictors of overall mortality in lung cancer patients. Increased serum SOD1 concentration per 1 pg/mL and SOD2 per 1 ng/mL were significantly associated with a 0.4 and 19% higher HR, respectively, for all-cause mortality in lung cancer patients.

The strongest predictive factor of all-cause mortality in lung cancer patients was an advanced clinical stage of disease; patients with stage IV disease had a 4.8 times higher hazard ratio (HR) than patients with stage I disease. A platelet count >400 × 10^3^ cells/µL was the strongest biochemical factor predicting mortality (HR = 2.26). Among parameters related to inflammation, GPS > 0 (HR = 1.59) was the strongest factor predicting mortality, followed by CRP > 10 mg/dL (HR = 1.55).

Table 4 presents two significant models of multiple Cox regression analysis concerning the prediction of mortality in the lung cancer group. The models confirmed that elevated serum SOD1 and SOD2 concentrations significantly increased the HR of all-cause mortality in lung cancer patients. Moreover, clinical stages III and IV were more strongly associated with mortality (HR = 2.55 for stage IV in model II compared to HR = 3.25 for stage III in model I) than SOD1 and SOD2 concentrations (HR = 1.005 for increased serum SOD1 per 1 pg/mL and HR = 1.30 for increased serum SOD2 per 1 ng/L). Again, models built with serum total SOD activity did not reveal any significant associations for predicting all-cause mortality in lung cancer patients.

The follow-up times and incidence of all-cause death in lung cancer patients by serum total SOD activity and SOD1/2 concentrations are presented in Table 5. Most importantly, serum SOD1 concentrations above the median displayed the highest incidence rate (17.01 per 1000 person months), while serum SOD1 concentrations below the median showed the lowest incidence rate (9.89 per 1000 person months).

**Figure 1:**
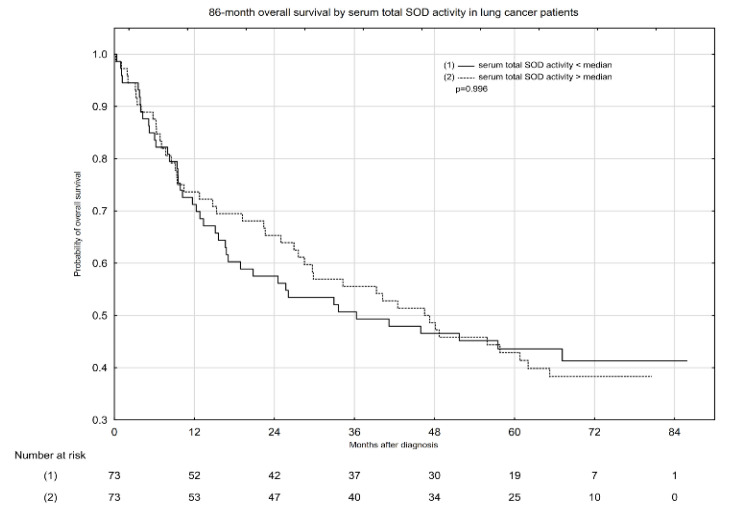
Eighty-six-month overall survival by serum total SOD activity in lung cancer patients.

**Figure 2:**
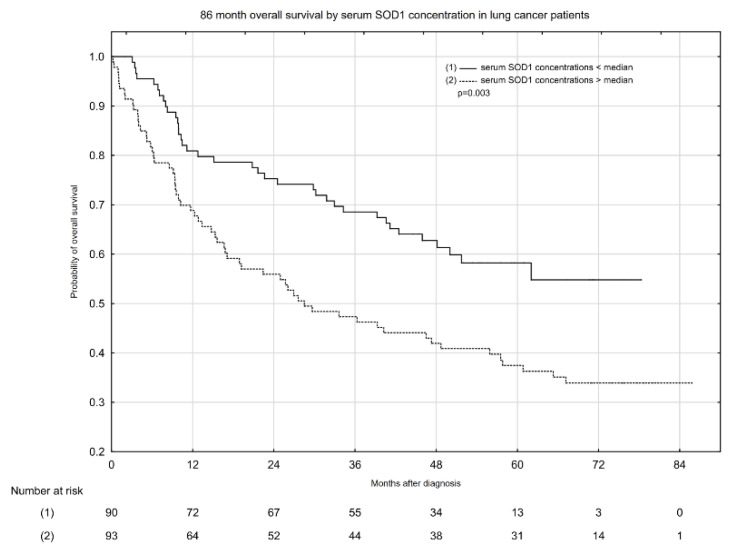
Eighty-six-month overall survival by serum SOD1 concentration in lung cancer patients.

**Figure 3:**
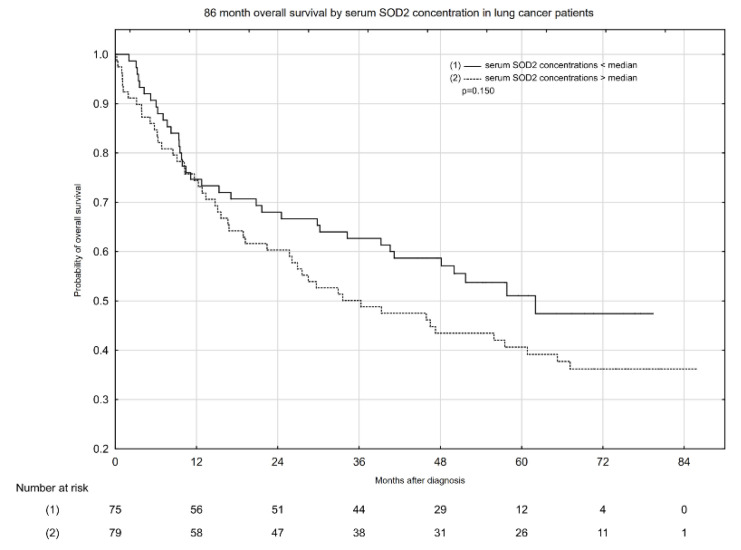
Eighty-six-month overall survival by serum SOD2 concentration in lung cancer patients.

Kaplan–Meier overall survival (OS) estimates according to median serum total SOD activity and serum SOD1 and SOD2 concentrations are presented in Figure 1, Figure 2 and Figure 3. Serum total SOD activity and SOD2 concentration below and above the median had an insignificant effect on the probability of 5-year OS (Figure 1 and Figure 3, respectively). Conversely, serum SOD1 concentration below the median significantly increased the probability of 5-year OS, from ~38 to 59% (Figure 2). 

## 3. Discussion

To the best of our knowledge, this is the first prospective cohort study with a long-term follow-up time investigating the diagnostic and predictive value of serum SOD enzymes in lung cancer patients. The sole previous study on the predictive value of SOD-related parameters in lung cancer was based on exhaled breath condensate total SOD activity, and was measured in a small group of 40 lung cancer patients with a short follow-up time of 36 months [26]. Although the cited research was performed on different test material, its results were auspicious, indicating a need for further study. Moreover, most previous studies concerning SOD in lung cancer focused only on one or two parameters: total SOD [26,27], or SOD1 and SOD2 concentrations [9,10,19]. In our study we took into account concentrations of SOD1 and SOD2, as well as activity of total SOD in serum. Serum is a biological material that is routinely and easily available from every patient and in every laboratory; therefore, the clinical utility of this material seems to be greater than that of other materials. Most of the previous studies concerning SOD activity and concentrations were performed on lung tumor tissues [9,10,28] or red and white blood cells [18,19,27]. Therefore, it is also very difficult to compare the aforementioned studies with one another, because the variation in the findings may be the result of the different test materials used. Our findings confirm the first study’s hypothesis to a good extent: SOD1 and SOD2 concentrations were higher in lung cancer patients than in the control group. However, serum total SOD activity did not vary significantly between patients with lung cancer and the control group. Inflammatory status and oxidative stress have been recognized as important parts of the pathogenesis of lung malignancies; therefore, changes in serum total SOD activity could be hypothesized [11,13]. Previous studies found that total SOD activity was significantly increased in lung tumor tissues [9], while in the study performed by Ho et al. [27], total SOD activity in erythrocytes was decreased in lung cancer patients compared to the control group, and significantly lower activity was also associated with the presence of clinically evident metastatic (stage IV) disease [27]. Meanwhile, in a study on colorectal cancer, total SOD levels in erythrocytes were significantly increased in patients compared to healthy controls [28]. Similarly, in breast cancer, serum SOD activity showed significant elevation in cancer patients compared to the control group, but irrespective of clinical stage [29]. The aforementioned differences in terms of changes in total SOD activity may result from multifaceted mechanisms involving interactions between carcinogenesis (as the main disturbance) and other background variables. More advanced stages of cancer may be associated with greater alterations in the systemic antioxidant profile [10]. These disturbances play critical roles in maintaining DNA stability and integrity by eliminating superoxide anions to prevent carcinogenesis [30,31]. As previously shown, lung cancer is strongly associated with redox status disturbances [32]; reactive oxygen species (ROS) are formed in excess by the lung tumor tissue, and enter the bloodstream, disrupting the systemic oxidative homeostasis. Additionally, in our study of lung cancer patients, a significantly higher number were former smokers and the link between cigarette smoking and redox imbalance is widely known [6,7,8]. Therefore, the above presented result remains unclear, and requires further study—especially regarding the mechanisms and background factors implicated in serum SOD activity during carcinogenesis. In the context of our results, it is noteworthy that in the study of Ho et al., where blood glutathione peroxidase (GPx) and catalase (CAT) activities were examined, GPx activity was significantly higher in lung cancer patients than in the control group [27]. The increased erythrocyte GPx activity in patients with lung cancer could suggest an increased breakdown of hydrogen peroxide in the blood, which may help in counteracting the accumulation of hydrogen peroxide in lung tumors [16]. Hydrogen peroxide has been shown to exert a stimulatory effect on SOD activity [27]. Therefore, the unaffected serum SOD activity in lung cancer patients may be a result of enhanced GPx-dependent reduction in hydrogen peroxide levels. The proposed associations in the antioxidant system in lung cancer remain speculative, and require further research, including assessment of GPx and CAT activity. 

In addition to the unchanged serum SOD activity in patients with lung cancer, we also could not confirm the prognostic value of this biochemical marker with regard to mortality. Ito et al. [25] conducted a population-based follow-up study of rural Japanese inhabitants, and found that serum SOD activity may be useful as a biomarker of protective effects against mortality from cancer. However, in another study of the Japanese population, a slight positive association was found between serum SOD levels and the risk of all-cause mortality in cancer [33]. Additionally, in a study performed on a Chinese cohort, increased SOD activity was independently associated with lower all-cause mortality in older women, but not in men [17]. However, we did not evaluate sex and age differences in predicting all-cause mortality with SOD-related parameters in this study, but this may be a good point of reference for future research. 

A survey of SOD isoform concentrations in the sera of lung cancer patients revealed significant alterations in both SOD1 and SOD2 levels in comparison with control subjects. Inflammation and oxidative disturbances might play key roles in promoting cancer progression and metastasis, because their mediators increase vascular permeability, facilitate cancer cell infiltration, and contribute to cancer cell adhesion to the endothelium and stromal invasion at metastatic sites [10]. In particular, increased SOD2 activity was shown to facilitate cell migration and tumor invasiveness in other investigations [21,22]. On the other hand, SOD1 and SOD2 are essential to cellular health, protecting bodily cells from excessive reactive oxygen species, free radicals, and other harmful agents that promote aging or cell death [21,22]. Our results on SOD1 and SOD2 concentrations are different to the data presented by Margaret et al. [19], but in the latter study the samples were taken from leukocyte cells. On the other hand, the data presented by Kaynar et al. [18] showed that the erythrocyte SOD1 activity was significantly higher in patients with lung cancer than in the control group and, interestingly, significantly higher in stage III–IV than stage I–II. Warsinggih et al. [30] observed significant differences in serum SOD1 activity with respect to colorectal cancer stages, where higher concentrations of SOD1 enzymes were recorded in advanced disease (stages III and IV). However, in our study, we found no associations between concentrations of serum SOD isoforms and the clinical stage of the disease (data are shown in Table S1). 

The ongoing oxidative and inflammatory disturbances in our cohort may also be demonstrated by the generally lower concentrations of albumin and higher CRP and ceruloplasmin concentrations in lung cancer patients compared to the control group. Albumin is a strong antioxidant of human extracellular fluids, with many different activities against reactive oxygen species [34], while CRP is a biomarker used to clinically measure systemic inflammation [35]. The significantly higher percentage of patients with lung cancer than control subjects with increased GPS scores reflect the alterations of these parameters in the disease. The GPS is one of the most extensively validated systemic-inflammation-based prognostic scores, and may therefore be used in the routine clinical assessment of patients with cancer [36]. The meta-analysis carried out by Jin et al. [37] showed that GPS may have prognostic value in lung cancer, and revealed a significant association between elevated GPS and poorer overall survival. Furthermore, in our study, according to univariate Cox regression, both GPS (>zero vs. zero) and CRP concentrations (>10 vs. ≤10 mg/dL) were shown to have predictive value with regard to all-cause mortality in lung cancer patients, increasing the risk of all-cause mortality in lung cancer patients by 59 and 55%, respectively. The disturbances in platelet count observed in the studied group might also be attributable to inflammatory processes, along with the progression of the underlying disease. According to the meta-analysis, reactive thrombocytosis, which often manifests in carcinogenesis—especially at advanced stages—may predict overall survival in various types of cancer, including lung cancer [5]. In our study, we found that platelet counts >400 × 10^3^ cells/µL increased the risk of all-cause mortality by 126% when compared to normal platelet counts. 

The hypotheses concerning the implications of disturbances in serum total SOD activity, as well as in SOD1 and SOD2 concentrations, with respect to biochemical variables and clinical data were not confirmed in our prospective cohort study, with the exception of the significantly lower concentrations of ceruloplasmin in lung cancer patients with SOD1 concentrations lower than the median. In addition, ceruloplasmin concentrations were significantly higher in lung cancer patients than in the control group. Copper (Cu) is one of the two cofactors in SOD1, and also plays an important role in ceruloplasmin activity, which is a serum ferroxidase [38]. Ceruloplasmin also contains more than 95% of the copper found in plasma [39]. Higher concentrations of SOD1 and ceruloplasmin may indicate an increased Cu demand—and, therefore, circulation of Cu—in patients with lung cancer. In addition, to meet the metabolic demands of the growth and development of cancer cells, higher Cu concentrations are also needed, especially with regard to the Warburg effect [40]. Interestingly, in a previous prospective cohort study, we found that Cu status in lung cancer patients was disrupted mainly via increased circulatory Cu, with concomitant decreased Zn concentration. This was reflected in significantly higher serum and whole blood Cu:Zn ratios in lung cancer patients compared to control subjects [6]. Most importantly, abnormal circulating Cu and Zn concentrations may contribute to carcinogenesis as a result of DNA damage, lipid peroxidation, protein modification, and other effects [41]. It could, for example, be a result of increased SOD1 and ceruloplasmin activity. However, further analyses are required in order to confirm the suggested association of ceruloplasmin, SOD1 concentration, and Cu homeostasis.

Most importantly, our analysis indicated that the serum SOD1 and SOD2 concentrations are good prognostic parameters of all-cause mortality in lung cancer. An increased concentration of serum SOD1 per 1 pg/mL increases the risk of all-cause mortality by 0.4%, while elevated serum SOD2 concentrations per 1 ng/mL increase the mortality risk by 19%. Additionally, the cutoff values of serum SOD1 and SOD2 concentrations that best differentiated survival and non-survival patients were 187.59 pg/Ml and 2.30 ng/mL, respectively. In order to more precisely estimate the predictive power of SOD1 and SOD2 concentrations, we adjusted the hazard ratios for the other clinical and biochemical data measured. According to multiple Cox regression models predicting lung cancer mortality, only serum concentrations of SOD1 and SOD2 and advanced clinical (III–IV) stages of disease remained statistically significant. Additionally, in our study, we found no associations between disease stage and serum total SOD activity or SOD1 and SOD2 concentrations. 

The serum SOD1 concentration appears to be better than the SOD2 concentration for the prediction of all-cause mortality in lung cancer, as demonstrated by our Kaplan–Meier overall survival estimates. Only serum SOD1 concentrations below the median significantly increased the probability of 5-year overall survival (by ca. 20%). Moreover, this study found that serum SOD1 concentrations above the median predicted the highest incidence of mortality (17.01 per 1000 person months), while serum SOD1 concentrations below the median had the lowest incidence rate (9.89 per 1000 person months). 

To the best of our knowledge, this is the first prospective cohort study to show the association of serum total SOD activity and SOD1/2 concentrations with lung cancer prognosis in pretreatment patients, taking into consideration a multifaceted background including clinical stage, comorbidities, and other clinical, biochemical, and sociodemographic data. Moreover, in this study, the cutoff values of serum SOD1 and SOD2 concentrations significantly differentiated lung cancer patients from the control group (SOD1), and predicted mortality in the studied group of lung cancer patients (SOD1 and SOD2). An additional advantage of this study was its long-term follow-up of 86 months.

Several limitations of this study should be acknowledged. Firstly, this was a relatively small prospective cohort study (n = 190 lung cancer cases) from a single center. Therefore, the present results may not be representative of the general population of lung cancer patients. In addition, using a retrospective questionnaire for the assessment of dietary intake might have produced response bias due to memory gaps and social-desirability bias of the participants. Furthermore, although we performed our analysis using multivariate Cox proportional hazards analysis, residual confounding by other unmeasured or unknown factors remains possible. In this study, it was not possible to control all factors influencing serum total SOD activity or SOD1 and SOD2 concentrations—for example, diet, lifestyle, and clinical parameters—therefore, further studies should be performed in a more homogenous group of lung cancer patients, or analyses should also be adjusted for the aforementioned data in order to better evaluate the impact of circulating enzymes on the prediction of mortality in lung cancer patients. 

## 4. Materials and Methods

### 4.1. Study Design and Protocol

This study was designed to evaluate the following hypotheses: (1) serum SOD activity and SOD1/2 concentrations are changed in lung cancer patients; (2) these alterations are related to both biochemical variables (albumin, CRP, ceruloplasmin, Hgb, platelets, creatinine, glucose concentrations, NLR, eGFR, and phosphatase alkaline activity) and clinical data (clinical stage of disease, type of lung cancer, presence of CVD, COPD, DM, anemia, reactive thrombocytosis, systemic inflammatory state, and elevated GPS), and (3) serum SOD activity and SOD1/2 concentrations can be used to predict all-cause mortality (endpoints) in patients with lung cancer.

### 4.2. Subjects

A total of 242 participants were enrolled in the study. Newly diagnosed lung cancer patients (n = 190) were recruited from the Lower Silesian Centre of Lung Diseases between June 2013 and December 2016 after confirmed diagnosis and any oncological treatment. All lung cancer diagnoses were confirmed by histopathological examination after surgery or bronchofiberoscopy. The clinical stage of disease and metastases were evaluated based on chest computed tomography (CT), positron emission tomography (PET)–CT, and ultrasonography of the abdominal cavity. CT/MRI (magnetic resonance imaging) of the central nervous system and bone scintigraphy were performed if necessary. In cases of enlarged lymph nodes of the mediastinum, endobronchial ultrasound transbronchial needle aspiration (EBUS-TBNA) was performed. Negative EBUS results were verified by mediastinoscopy.

Exclusion criteria were as follows: mental illness that could make it difficult to obtain reliable results, other malignant diseases, or prior oncological therapy for any malignant disease.

The control group (n = 52) consisted of healthy people recruited from the Wroclaw Universities of the Third Age and public offices. Exclusion criteria for the control group were as follows: cancers, metabolic disturbances, other proinflammatory diseases, neurological disorders, and mental health issues.

The study protocol was approved by the Ethics Commission of Wroclaw Medical University (approval No. 540/2013), and the study was conducted in accordance with the principles expressed in the Declaration of Helsinki. All participants provided written informed consent prior to the research.

#### Sociodemographic Characteristics

The sociodemographic characteristics of the lung cancer patients and control subjects were identified based on the following criteria: sex, age, level of education, smoking status, and alcohol consumption. In terms of smoking, all participants were classified as never (never or >1 year smoking cessation), previous (≤1 year smoking cessation), or current (active smoking or ≤2 weeks smoking cessation). Cigarette consumption was assessed only for current smokers, and they were divided into the following groups: sporadic smokers, <5 cigarettes per day, 5–20 cigarettes per day, or >20 cigarettes per day. Participants were classified as alcohol consumers (at least 10 g of ethanol/week) or non-consumers. Alcohol consumers were divided into groups by their intake: 0.5–2, 3–5, 6–10, or ≥11 units of alcohol per week. 

### 4.3. Blood Sample Collection

Blood samples from lung cancer patients and control subjects were collected after 10–12 h overnight fasting, and were drawn after 15 min rest in the sitting position. In the case of patients, samples were taken the day after hospital admission. Whole blood, plasma, and serum samples were used to perform the laboratory tests. Tubes containing an anticoagulant were used to obtain the whole blood and plasma. In order to separate the plasma, the samples were centrifuged at 2000× *g* for 5 min immediately after collection. However, in order to obtain serum, tubes with clotting activator were used; after 30 min of clotting at room temperature, the samples were centrifuged at 2000× *g* for 10 min, and the separated serum was aliquoted and stored at −80 °C until laboratory analyses were performed.

#### 4.3.1. Biochemical and Clinical Characteristics

Patients were identified in terms of the clinical stage of disease, type of lung cancer, type of treatments, and presence of the following comorbidities: DM type 1 or 2, CVD, and/or COPD. Laboratory analysis was conducted to assess the clinical condition of lung cancer patients. Whole blood Hgb concentrations, platelet counts, and neutrophil and lymphocyte concentrations were determined using a Sysmex IXP 1080i automatic hematology analyzer. Anemia was diagnosed as Hgb concentration <13.7 g/dL in men under 60 years old, <13.2 g/dL in men over 60 years old, and <12.2 g/dL in all women, regardless of age [42]. The reference range of platelet counts was 150–400 × 10^3^ cells/µL [43].

Measurement of serum creatinine concentration and phosphatase alkaline activity, as well as eGFR calculation, were performed using a Cobas Roche Integra 400+. Plasma glucose concentration was measured using an SAM biochemical analyzer. All biochemical analyses listed above were performed routinely in the hospital on the day of sample collection. Patients were divided into 3 groups by fasting glucose concentration: normal (<100 mg/dL), impaired (100–125 mg/dL), and diabetic (≥126 mg/dL) [44]. The normal eGFR rate was defined as ≥90 mL/min/1.73 m^2^, while the normal range of serum creatinine concentration was 0.7–1.2 mg/dL [45]. 

##### Determinations of Serum Total SOD Activity and Serum SOD1 and SOD2 Concentrations

Serum total SOD activity was measured with the Superoxide Dismutase Assay Kit (Cayman Chemical Company, Ann Arbor, MI, USA, REF: 706002). The methodology was based on the reaction in which xanthine oxidase converts xanthine and O_2_ into uric acid and H_2_O_2_ to produce superoxide ions. The superoxide ions formed in this reaction convert the tetrazolium salt into formazan dye. Simultaneously, the superoxide dismutase from the analyzed serum samples decomposes the superoxide ions in the reaction mixture, and thus reduces the amount of formed formazan dye. The results were measured spectrophotometrically, and expressed as U/mL, where 1 unit of SOD is defined as the enzyme concentration required to achieve 50% dismutation of the formed superoxide radicals. Samples were diluted, and the dilution ratio was taken into account in the final calculations, in accordance with the manufacturer’s instructions.

SOD1 concentration in serum samples was measured using an ELISA kit (Cloud-Clone Corp., Houston, TX, USA. REF: SEB960Hu) while SOD2 concentration in serum samples was measured using another ELISA kit (Cloud-Clone Corp., Houston, TX, USA, REF: SEB083Hu). Specifically, the microplates were precoated with antibodies that specifically bind to either SOD1 or SOD2. First the standards or serum samples containing SOD1 or SOD2 antigens, and then the secondary biotin-conjugated antibody specific to SOD1 or SOD2, were added to the appropriate microplate’s wells. In the next step, avidin conjugated to a horseradish peroxidase complex was bound to the biotinylated antibody complexed with the immobilized SOD1 or SOD2. After adding the TMB chromogen to the mixture, the enzyme bound to avidin caused its conversion to a colored product. The enzyme–substrate reaction was terminated in a timely manner by the addition of sulfuric acid solution, and the absorbance was measured at 450 nm. The results were calculated from a standard 4-parameter logistic curve, taking into account the dilution of the samples, and expressed in pg/mL and ng/mL for SOD1 and SOD2 concentrations, respectively.

The total SOD activity and SOD1/2 concentrations were determined using a multifunctional microplate reader with data management software (MR-96, Mindray, Shenzhen, China).

##### Determination of Serum Ceruloplasmin, Albumin, and CRP

Levels of ceruloplasmin in serum samples were measured by the immunoprecipitation method. Antihuman ceruloplasmin antibodies were added in excess to the serum samples. The increase in absorbance caused by immunoprecipitation was measured at 340 nm at the endpoint of the reaction. The results were calculated from the calibration curve and expressed in g/L. The assigned values of the calibrators were made traceable to the European Reference Material ERM-DA470.

The albumin concentrations were measured via the colorimetric method. The albumin reacted with bromocresol green, and a colored product was formed. The absorbance was measured at 600 nm at the endpoint of the reaction. The results were calculated from the calibration curve and expressed in g/dL. The assigned values of the calibrators were made traceable to the European Reference Material EMR-DA470k. 

Levels of CRP in serum samples were measured via the immunoprecipitation method, with the high-sensitivity application. Antihuman CRP antibodies binding to the polystyrene particles were added to the serum samples, and the rate of absorbance increase caused by immunoprecipitation was measured bichromatically, at 800 and 505 nm. The results were calculated from the calibration curve and expressed in mg/L. The assigned values of the calibrators were made traceable to the IFCC reference material ERM^®^ -DA474.

Measurement of ceruloplasmin, albumin, and CRP concentrations was performed using an auto-analyzer Konelab 20i (Thermo Scientific, Waltham, MA, USA) with serum samples stored at −80 °C.

#### 4.3.2. Glasgow Prognostic Score

The GPS is a cumulative prognostic score based on the systemic inflammatory response (serum CRP concentration) and albumin concentration [36]. The scale of GPS (0–2) is defined as follows: patients with both an elevated CRP concentration (>10 mg/l) and hypoalbuminemia (<35 g/dL) are allocated a score of 2; patients in whom only one of these biochemical disturbances are present are allocated a score of 1. Patients in whom neither of these disturbances is present are allocated a score of 0. 

### 4.4. Statistical Analysis

All data are presented as median values (Q1–Q3) and were analyzed using Statistica 13.3, PL (StatSoft, Tulsa, OK, USA). We used the Shapiro–Wilk test to analyze the normality of the data distribution within the groups of lung cancer and control subjects and subgroups of lung cancer patients. To evaluate the differences in continuous data between lung cancer patients and the control group, and between subgroups of lung cancer patients, Student’s *t*-test (for parametric data distribution) or the Mann–Whitney U test (for non-parametric data distribution) were performed. A chi-squared test was used to determine significant differences in the distribution of categorical variables of characteristics between groups or subgroups.

We followed the lung cancer study population from the date of diagnosis until death or 1 September 2020, with a median (range) follow-up time for this study of 45.42 (0.23–85.81) months. Death was established based on data from the Registry Office. Lung cancer patients were linked to the data statistics registry records using a unique 11-digit identification number (PESEL) and their first and last names. Since causes of death were not available, only all-cause mortality was analyzed.

Receiver operating characteristic (ROC) curves, areas under the ROC curves (AUCs), and 95% confidence intervals for AUCs, along with Youden’s index, were calculated to determine cutoff values of serum total SOD activity and serum SOD1/2 concentrations for the diagnosis of lung cancer, as well as the identification of clinical stage IV, and prediction of mortality in lung cancer patients, using ROC curves.

We evaluated the relationship between serum total SOD activity, serum SOD1 and SOD2 concentrations, and all-cause mortality using Cox proportional hazards analysis. First, we built univariate Cox regression models to evaluate the significance in predicting mortality of each parameter related to biochemical (continuous variables), clinical (categorical variables), sociodemographic (categorical variables), as well as for the following SOD-related parameters: serum total SOD activity per 1 U/mL, serum SOD1 concentration per 1 pg/mL, and serum SOD2 concentration per 1 ng/mL. Then, for each SOD-related parameter (serum total SOD activity and serum SOD1 and SOD2 concentrations), we built a model of multivariate Cox regression analysis. The models were adjusted using the following parameters: albumin (continuous), DM (yes/no), clinical stage (I/II/III/IV), platelet count (<150/150–400/>400 10^3^ cells/µL), NLR (<2.67 vs. ≥2.67 arbitrary unit), ceruloplasmin (<0.266 vs. ≥0.266 g/L), CRP (≤10 vs. >10 mg/dL), and GPS (arbitrary unit). The stability of the model was certified by using a likelihood ratio step-forward fitting procedure. Additionally, overall 86-month survival according to serum total SOD activity and serum SOD1 and SOD2 concentrations was demonstrated using Kaplan–Meier curves. The relationships between each of these variables (presented as categorical data: < and ≥ median of concentration for the entire lung cancer patient group) and overall survival were assessed via log-rank test. For all statistical procedures, the significance level was considered to be <0.05.

## 5. Conclusions

In conclusion, higher SOD1 and SOD2 concentrations were shown to positively affect the risk of all-cause mortality in lung cancer patients, but the serum SOD1 concentration appears to be a better predictor than the serum SOD2 concentration. The results obtained encourage further in-depth study of the role of serum total SOD activity in lung carcinogenesis, and its potential utility in predicting lung cancer mortality. The findings of this study suggest that serum SOD1 concentration could be included in the routine clinical characteristics of lung cancer patients as an additional diagnostic and prognostic biomarker; however, this still required corroboration by further, more detailed studies with larger cohorts. Additionally, the results of our study are valuable also in the context of future studies on, e.g., treatment development which includes redox status modulations, because lung carcinogenesis and its progression is directly linked with redox imbalance.

## Figures and Tables

**Table 1:** Baseline characteristic of lung cancer patients and the control group.

Parameters	n	Lung Cancer Patients	n	Control Group	*p*
**Sociodemographic Factors**					
**Sex, F/M (%, n)**	190	43.7/56.3 (83/107)	52	40.4/59.6 (21/31)	0.670
**Age, years (median (Q1–Q3))**	186	65.0 (60.0–71.0)	52	63.0 (50.0–68.0)	0.005
**Education: Primary/vocational/high school/college (%, n)**	176	14.7/31.6/36.7/16.4 (26/56/65/29)	52	7.7/19.2/44.2/28.9 (4/10/23/15)	0.100
**Smoking status: Never/previous/current (%, n)**	177	23.2/48.6/28.2 (41/86/50)	52	53.8/19.2/26.9 (28/10/14)	<0.001
**Passive smoking: Yes/no (%, n)**	177	22.6/77.4 (40/137)	52	23.1/76.9 (12/40)	0.942
**Number of cigarettes per day:** **Sporadically/<5/5–20/>20 (%, n)**	50	2.0/22.0/70.0/6.0 (1/11/35/3)	14	21.4/21.4/50.0/7.2 (3/3/7/1)	0.063
**Alcohol consumption: Yes/no (%, n)**	177	50.3/49.7 (88/89)	52	76.9/23.1 (40/12)	<0.001
**Number of alcohol portion (10 g of ethanol) per week:** **0.5–2/3–5/6–10/** **≥** **11 (%, n)**	88	44.3/26.1/20.4/9.1 (39/23/18/8)	38	50.0/15.8/28.9/5.3 (19/6/11/2)	0.433
**Biochemical and clinical characteristics**					
**Total SOD, U/mL (median (Q1–Q3))**	187	1.08 (0.85–1.27)	52	1.06 (0.90–1.30)	0.932
**SOD1, pg/mL (median (Q1–Q3))**	187	218.9 (133.5–283.6)	52	141.0 (98.0–225.6)	<0.001
**SOD2, ng/mL (median (Q1–Q3))**	187	1.30 (0.53–2.31)	34	0.78 (0.22–1.49)	<0.001
**Albumin, g/dl (median (Q1–Q3))**	176	3.86 (3.55–4.17)	50	4.27 (4.12–4.46)	<0.001
**Albumin: <3.5/≥3.5, g/dL (%, n)**	176	18.2/81.8 (32/144)	50	4.0/96.0 (2/48)	0.013
**CRP, mg/L (median (Q1–Q3))**	176	11.24 (3.13–108.11)	50	2.26 (0.92–3.99)	<0.001
**CRP: ≤10/>10, mg/L (%, n)**	176	46.6/53.4 (82/94)	50	94.0/6.0 (47/3)	<0.001
**GPS: 0/1/2, arbitrary unit (%, n)**	176	41.5/45.5/13.1 (73/80/23)	50	90.0/10.0/0.0 (45/5/0)	<0.001
**Ceruloplasmin, g/l (median (Q1–Q3))**	175	0.26 (0.22–0.31)	50	0.21 (0.19–0.24)	<0.001
**Clinical stage of disease: I/II/III/IV (%, n)**	152	41.4/18.4/16.4/23.7 (63/28/25/36)	NA		
**Type of lung cancer: NSCLC/SCLC/carcinoid (%, n)**	166	94.0/5.4/0.6 (156/9/1)			
**Type of treatment: chemotherapy/radiotherapy/surgery (%,n)**	161	32.9/4.3/70.2 (53/7/113)			
**CVD: Yes/no (%, n)**	150	44.7/55.3 (67/83)			
**COPD: Yes/no (%, n)**	152	13.8/86.2 (21/131)			
**DM: Yes/no (%, n)**	153	17.6/82.4 (27/126)			
**Hgb, g/dL (median (Q1–Q3))**	178	13.0 (11.8–14.1)			
**Anemia: Yes/no (%, n)**	178	39.9/60.1 (71/107)			
**Platelets, x 10^3^ cells/µL (median (Q1–Q3))**	178	256.0 (202.0–315.0)			
**Platelets: <150/150–400/>400, × 10^3^ cells/µL (%, n)**	178	10.7/82.6/6.7 (19/147/12)			
**NLR, arbitrary unit (median (Q1–Q3))**	122	2.5 (1.70–4.40)			
**Phosphatase alkaline, U/L (median (Q1–Q3))**	106	79.92 (63.22–91.89)			
**eGFR ≥ 90/< 90, mL/min/1.73 m^2^ (%, n)**	154	63.6/36.4 (98/56)			
**Creatinine, mg/dL (median (Q1–Q3))**	165	0.76 (0.64–0.9)			
**Creatinine: <0.7/0.7–1.2/>1.2, mg/dL (%, n)**	165	37.0/55.7/7.3 (61/92/12)			
**Glucose, mg/dL (median (Q1–Q3))**	126	101.0 (93.2–115.7)			
**Glucose <100/100–125/≥126, mg/dL (%, n)**	126	43.6/40.5/15.9 (55/51/20)			

F: female; M: male; SOD: total superoxide dismutase; SOD1: superoxide dismutase 1; SOD2: superoxide dismutase 2; CRP: C-reactive protein; GPS: Glasgow prognostic score; NSCLC: non-small-cell lung cancer; SCLC: small-cell lung cancer; CVD: cardiovascular disease; COPD: chronic obstructive pulmonary disease; DM: diabetes mellitus type 1 or 2; Hgb: hemoglobin; NLR: neutrophil:lymphocyte ratio; eGFR: estimated glomerular filtration rate; Q1: first quartile; Q3: third quartile.

**Table 2:** Cutoff values, areas under the ROC curve (95% CI), and Youden’s indices for parameters differentiating lung cancer patients vs. the control group, lung cancer clinical stages I–III vs. IV, and survival vs. non-survival lung cancer patients.

Parameter	Cutoff Value	AUC (95% CI)	Youden’s Index	*p*
**Lung cancer patients vs. control group**				
**Serum total SOD activity (U/mL)**	1.51	0.495 (0.385–0.605)	0.06	0.928
**Serum SOD1 (pg/mL)**	175.03	0.684 (0.608–0.759)	0.31	<0.001
**Serum SOD2 (ng/mL)**	0.23	0.469 (0.369–0.569)	0.12	0.546
**Lung cancer patients in clinical stage I–III vs. IV**				
**Serum total SOD activity (U/mL)**	1.47	0.461 (0.336–0.587)	0.07	0.546
**Serum SOD 1 (pg/mL)**	115.51	0.493 (0.385–0.602)	0.12	0.905
**Serum SOD 2 (ng/mL)**	1.66	0.506 (0.387–0.625)	0.09	0.926
**Survival vs. non-survival lung cancer patients**				
**Serum total SOD activity (U/mL)**	1.29	0.545 (0.451–0.638)	0.12	0.350
**Serum SOD1 (pg/mL)**	187.59	0.654 (0.576–0.733)	0.24	<0.001
**Serum SOD2 (ng/mL)**	2.30	0.618 (0.530–0.705)	0.19	0.008

SOD: superoxide dismutase; SOD1: superoxide dismutase 1; SOD2: superoxide dismutase 2; AUC: area under the ROC curve; CI: confidence interval.

**Table 3:** Univariate Cox regression models in predicting lung cancer mortality.

Risk Factor	Univariable Cox Regression Models		
	HR	95% CI HR	*p*
**SOD1 per 1 pg/mL**	1.004	1.002–1.006	<0.001
**SOD2 per 1 ng/mL**	1.19	1.09–1.30	<0.001
**Albumin per 1 g/dL**	0.62	0.45–0.84	0.002
**CRP > 10 mg/dL**	1.55	1.01–2.36	0.044
**GPS > 0, arbitrary unit**	1.59	1.03–2.45	0.037
**Ceruloplasmin ≥ 0.266 vs. < 0.266 g/L**	1.55	1.02–2.36	0.041
**Clinical stage: III vs. I**	3.01	1.61–5.65	<0.001
**Clinical stage: IV vs. I**	4.84	2.79–8.41	<0.001
**DM: Yes vs. No**	1.80	1.09–2.97	0.022
**Platelets > 400 × 10^3^ vs. 150–400 × 10^3^ cells/µL**	2.26	1.30–3.95	0.004
**NLR ≥ 2.67 vs. < 2.67 arbitrary unit**	1.85	1.18–2.91	0.007
**Phosphatase alkaline per 1 U/L**	1.008	1.001–1.015	0.019
**Glucose per 1 mg/dL**	1.007	1.0005–1.013	0.035

SOD1: superoxide dismutase 1; SOD2: superoxide dismutase 2; CRP: C-reactive protein; GPS: Glasgow prognostic score; DM: diabetes mellitus; NLR: neutrophil:lymphocyte ratio; HR: hazard ratio; CI: confidence interval.

**Table 4:** Multiple Cox regression models predicting lung cancer mortality.

Model	Parameters	HR	95% CI HR	*p*
**I (for serum SOD1 concentration)**	Serum SOD1 per 1 pg/mL	1.005	1.002–1.008	<0.001
	Clinical stage IV vs. I	3.22	1.61–6.44	<0.001
	Clinical stage III vs. I	3.25	1.53–6.90	0.002
	Clinical stage II vs. I	0.61	0.22–1.72	0.350
**II (for serum SOD2 concentration)**	Serum SOD2 per 1 ng/mL	1.30	1.09–1.56	0.005
	Clinical stage IV vs. I	2.55	1.22–5.35	0.013
	Clinical stage III vs. I	3.03	1.40–6.56	0.005
	Clinical stage II vs. I	0.76	0.27–2.15	0.602

SOD1: superoxide dismutase 1; SOD2: superoxide dismutase 2; HR: hazard ratio; CI: confidence interval. Models I and II were adjusted using the following parameters: albumin (continuous), DM (yes/no), clinical stage (I/II/III/IV), platelet count (<150/150–400/>400 × 10^3^ cells/µL), NLR (<2.67 vs. ≥2.67 arbitrary unit), ceruloplasmin (<0.266 vs. ≥0.266 g/L), CRP (≤10 vs. >10 mg/dL), and GPS (arbitrary unit).

**Table 5:** Follow-up times and incidence of all-cause death in lung cancer patients by serum SOD total activity and serum SOD1 and SOD2 concentrations.

Parameters	Person Months	Number of Events	Incidence Rates *	Median (Range) of Follow Up Time
**Overall**	7434.35	100	13.45	45.42 (0.23–85.81)
**Serum total SOD activity < median**	2847.94	42	14.75	36.30 (0.39–85.81)
**Serum total SOD activity > median**	3080.32	44	14.28	46.52 (0.23–80.52)
**Serum SOD1 concentration < median**	3842.83	38	9.89	47.51 (1.94–78.35)
**Serum SOD1 concentration > median**	3586.13	61	17.01	28.50 (0.23–85.81)
**Serum SOD2 concentration < median**	3052.47	37	12.12	48.13 (2.04–79.40)
**Serum SOD2 concentration > median**	3129.96	49	15.66	33.60 (0.23–85.81)

SOD: superoxide dismutase; SOD1: superoxide dismutase 1; SOD2: superoxide dismutase 2; *: per 1000 person months; event (endpoint) was defined as all-cause mortality in lung cancer patients.

## Data Availability

Data is contained within the article and Supplementary Materials.

## Supplementary Materials

The following are available online at https://www.mdpi.com/article/10.3390/ph14111067/s1. Table S1: Biochemical and clinical data of lung cancer patients by serum total SOD activity and serum SOD1/2 concentrations.
