# Supplementary material for: Serum Total SOD Activity and SOD1/2 Concentrations in Predicting All-Cause Mortality in Lung Cancer Patients
Source: Pharmaceuticals (Basel). 2021 Oct 21;14(11):1067. doi: 10.3390/ph14111067 (PMC8622563; doi:10.3390/ph14111067)
Supplement: Supplementary file 1 [file pharmaceuticals-14-01067-s001.zip › pharmaceuticals-1402952-supplementary.pdf]

Table S1. Biochemical and clinical data of lung cancer patients by serum total SOD activity and serum SOD1, SOD2 concentrations

Variables	<median total SOD activity	>median SOD activity	p	<median SOD1 concentration	>median SOD1 concentration	p	<median SOD2 concentration	>median SOD2 concentration	p
Albumin, g/dl (median (Q1-Q3))	3.84 (3.64-4.19)	3.92 (3.5-4.12)	0.783	3.95 (3.55-4.18)	3.82 (3.59-4.07)	0.401	3.93 (3.58-4.24)	3.83 (3.61-4.15)	0.716
Albumin <3.5/≥3.5 g/dL	13.0/87.0 (9/60)	22.1/77.9 (15/53)	0.165	20.0/80.0 (18/72)	16.3/83.7 (14/72)	0.522	15.8/84.2 (12/64)	13.9/86.1 (10/62)	0.745
CRP, mg/l (median (Q1-	10.96 (3.03-97.08)	11.46 (3.07-114.97)	0.998	11.07 (3.03-118,13)	11.96 (3.24-98.93)	0.662	11.46 (4.31-115-03)	10.80 (2.38-98.01)	0.364
CRP ≤10/ >10 mg/l (%, n)	46.4/53.6 (32/37)	47.1/52.9 (32/36)	0.936	47.8/52.2 (43/47)	45.4/54.6 (39/47)	0.747	47.4/52.6 (36/40)	48.6/51.4 (35/37)	0.880
GPS 0/1/2 arbitrary unit	43.5/46.4/10.1 (30/32/7)	39.7/45.6/14.7 (27/31/10)	0.706	43.3/41.1/15.6 (39/37/14)	39.5/50.0/10.5 (34/43/9)	0.409	42.1/47.4/10.5 (32/36/8)	44.4/45.8/9.7 (32/33/7)	0.956
Ceruloplasmin, g/l (median (Q1-	0.26 (0.22-0.31)	0.26 (0.23-0.32)	0.908	0.26 (0.21-0.30)	0.28 (0.23-0.33)	0.035	0.27 (0.22-0.31)	0.29 (0.23-0.33)	0.062
Ceruloplasmin <0.26/ >0.26 g/l	52.2/47.8 (36/33)	51.5/48.5 (35/33)	0.934	51.7/48.3 (46/43)	47.7/52.3 (41/45)	0.596	44.7/55.3 (34/42)	43.7/56.3 (31/40)	0.896
Clinical stage of disease: I/II/III/IV (%, n)	31.7/18.3/21.7/28.3 (19/11/13/17)	46.2/18.5/15.4/20.0 (30/12/10/13)	0.356	42.3/19.7/12.7/25.4 (30/14/9/18)	41.3/17.5/20.0/21.3 (33/14/16/17)	0.659	47.0/15.2/13.6/24.2 (31/10/9/16)	35.9/21.9/21.9/20.3 (23/14/14/13)	0.359
CVD: Yes/no (%, n)	35.1/64.9 (26/48)	32.4/67.6 (24/50)	0.728	38.3/61.7 (36/58)	33.0/67.0 (31/63)	0.446	41.8/58.2 (33/46)	27.9/72.2 (22/57)	0.066
COPD: Yes/no (%, n)	9.5/90.5 (7/67)	12.2/87.8 (9/65)	0.597	12.8/87.2 (12/82)	9.6/90.4 (9/85)	0.487	11.4/88.6 (9/70)	10.1/89.9 (8/71)	0.797
DM: Yes/no (%, n)	18.9/81.1 (14/60)	9.5/90.5 (7/67)	0.099	12.8/87.2 (12/82)	14.9/85.1 (14/80)	0.673	13.9/86.1 (11/68)	15.2/84.8 (12/67)	0.822

Hgb, g/dL median	13.2 (11.8-14.3)	12.9 (11.9 – 14.2)	0.617	13.0 (11,9-14.0)	13.0 (11.7 – 14.3)	0.800	13.0 (11.6 – 13.8)	13.0 (11.8 – 14.3)	0.540
Anemia: Yes/no (%,n)	41.1/58.9 (30/43)	40.3/59.7 (29/43)	0.920	38.1/61.9 (32/52)	41.9/58.1 (39/54)	0.603	39.7/60.3 (29/44)	42.9/57.1 (33/44)	0.697
Platelets, 10^3 cells/µL	266.0 (202.0-336.0)	246.5 (195-306.5)	0.256	253.5 (198.5-299.5)	259.0 (203.0-338.0)	0.284	259.0 (200.0-305.0)	261.0 (221.0 – 345.0)	0.405
Platelets <150/150-	2.7/84.9/12.3 (2/62/9)	9.7/79.2/11.1 (7/57/8)	0.219	8.3/85.7/6.0 (7/72/5)	5.4/80.7/14.0 (5/75/13)	0.284	6.9/ 84.9/8.2 (5/62/6)	16.9/ 76.6 / 6.5 (13/59/5)	0.405
NLR, arbitrary unit (median	2.65 (1.72-4.98)	2.71 (1.70-4.69)	0.911	2.47 (1.52-4.13)	2.67 (1.95-4.98)	0.207	2.61 (1.65 – 4.69)	2.60 (1.99-4.96)	0.621
NLR,<2.67/ ≥2.67 arbitrary	51.1/48.9 (24/23)	50.0/50.0 (22/22)	0.919	58.2/41.8 (39/28)	49.1/50.1 (27/28)	0.315	53.6/46.4 (30/26)	51.0/49.0 (25/24)	0.794
Alkaline Phosphatase	83.1 (62.3 – 97.6)	79.9 (63.2 – 88.0)	0.495	79.9 (74.1 – 88.4)	80.8 (61.0 – 95.8)	0.634	88.7 (71.9 – 99.8)	76.9 (61.1 – 85.3)	0.172
eGFR ≥90/<90 mL/min/1.73m²	59.3/40.7 (35/24)	67.2/31.3 (45/12)	0.377	67.1/32.9 (49/24)	60.5/38.3 (49/31)	0.477	66.7/33.3 (40/20)	62.5/36.1 (45/26)	0.608
Creatinine, mg/dL (median	0.78 (0.65-0.95)	0.77 (0.61-0.86)	0.374	0.74 (0.60-0.87)	0.77 (0.64-0.95)	0.327	0.76 (0.63-0.87)	0.73 (0.65-0.94)	0.544
Creatinine: <0.7/0.7-1.2/>1.2	36.9/55.4/7.7 (24/36/5)	33.3/60.9/5.8 (23/42/4)	0.789	41.8/50.6/7.6 (33/40/6)	32.9/60.0/7.1 (28/51/6)	0.327	39.4/56.1/4.6 (26/37/3)	37.0/54.8/8.2 (27/40/6)	0.544
Glucose, mg/dL (median (Q1-	102.8 (96.8-119.2)	100.4 (90.2 – 111.1)	0.167	101.0 (93.0-115.9)	101.7 (93.3-111.1)	0.947	105.7 (96.2-118.8)	99.0 (89.7 - 110.7)	0.056
Glucose <100.0/100.0-	40.4/38.5/21.2 (21/20/11)	45.8/45.8/8.5 (27/27/5)	0.164	45.5/36.4/18.2 (25/20/10)	42.9/44.3/12.9 (30/31/9)	0.578	38.3/42.6/19.2 (18/20/9)	50.8/36.1/13.1 (31/22/8)	0.402

SOD, total superoxide dismutase; SOD1, superoxide dismutase 1; SOD2, superoxide dismutase 2; CRP, C- reactive protein; GPS, Glasgow prognostic score; NSCLC, non-small cell lung cancer; SCLC, small cell lung cancer; CVD, cardiovascular disease; COPD, chronic obstructive pulmonary disease; DM, diabetes mellitus type 1 or 2; Hgb, hemoglobin; NLR, neutrophil:lymphocyte ratio; eGFR, estimated glomerular filtration rate; <SOD median – <1.08 U/ml; >SOD median - >1.08 U/ml; <SOD1 median – <218.9 pg/ml; >SOD 1median - >218.9 pg/ml; <SOD2 median – <1.30 ng/ml; >SOD2 median - >1.30 ng/ml;
